# Observations on the kinematic characteristics of the healthy side of the knee in stroke patients: A cross-sectional study

**DOI:** 10.1097/MD.0000000000031853

**Published:** 2022-11-18

**Authors:** JunWu Yu, Chen Wang, FaMing Yang

**Affiliations:** a Department of Rehabilitation, Ningbo College of Health Sciences, Ningbo, China; b School of Sport Medicine and Rehabilitation, Beijing Sport University, Beijing, China.

**Keywords:** healthy side, kinematic characteristics, knee joint, stroke

## Abstract

The abnormal gait of stroke patients not only severely limits the recovery of their walking ability, but also seriously affects their quality of daily life. Previous observational studies have focused too much on the observation of single degree of freedom and axial knee motion angles in stroke patients. Changes in the multi-degree of freedom and multi-axial joint angles of the knee have been less frequently observed, leading to somewhat limited conclusions. Therefore, the aim of this study was to use the Opti-knee motion test to analyze in real time the motion of the knee in all directions on the healthy side of stroke patients and to compare it with normal gait to provide a clinical basis for subsequent rehabilitation. In a cross-sectional study, 120 subjects (60 stroke patients were as the observation group and 60 healthy subjects as the control group) were studied. Both groups of subjects were tested for Opti-Knee tri-axial angles of motion of the healthy side of the knee, including flexion and extension, internal and external rotation, internal and external turning, anterior and posterior displacement, superior and inferior displacement, left and right displacement, maximum extension angle and maximum flexion angle. Compared with the control group, there were significant changes in the joint angles of flexion and extension, internal and external rotation, internal and external turning, maximum extension and maximum flexion of the knee on the healthy side in the observation group, and the differences were statistically significant [95%(37.22, 45.13), *P* = .01], [95%(9.51,13.67), *P* = .018], [95%(4.82,7.57), *P* = .049], [95%(4.12, 8.63), *P* = .019], [95%(51.68, 57.28), *P* = .0001]. However, there was no significant change in the angle of motion of the healthy side of the knee for anterior-posterior displacement, superior-inferior displacement and internal-external displacement in either group and the differences were not statistically significant [95%(1.16, 1.78), *P* = .72], [95%(0.85,1.32), *P* = .32], [95%(0.57, 0.88), *P* = .36]. This study confirms the importance of changes in the angle of motion of the knee on the side of the stroke patient in maintaining the stability of the knee joint. Therefore, their bilateral lower limb symmetry training should be paid attention to in the subsequent rehabilitation treatment.

## 1. Introduction

The stroke has become a major global health concern in recent years. Strokes are often classified as ischemic or hemorrhagic and are characterized by high recurrence, mortality and disability rates.^[1]^ According to insufficient statistics, about 1/5 of patients require further rehabilitation after a stroke and 1/3 of patients have a lifelong injury, which impacts a heavy financial burden on patients and their families.^[2]^

It has been found that as stroke patients move from the acute to the recovery phase, the muscles around the affected knee gradually regain strength. At the same time, increased muscle tone and contraction of the active and antagonist muscles during exercise prevents effective separation and valgus or extension of the knee joint, resulting in abnormal gait and increased dependent behavior, including slow walking speed, poor muscular endurance, uncoordinated gait and lessened balance, which decreases the patient’s capacity to perform daily activities.^[3]^ These abnormalities result in the common circle gait of stroke patients, which causes them to walk with excessive weight bearing on the right lower limb and has an impact on the recovery of walking ability in stroke patients.^[4]^

Previous studies have found that we have paid too much attention to the analysis of kinematic parameters of the lower limb on the healthy and affected side of stroke patients, such as gait frequency, gait speed, gait amplitude, gait cycle and stride length.^[5,6]^ Some studies have analyzed the flexion and extension angles of the hip, knee and ankle joints bilaterally in stroke patients using 3D gait as a reference for gait analysis.^[6]^ However, the joint motion angles of the lower limbs measured in these studies were only observed in a single axial direction, and there are fewer studies on the multidimensional and multi-axial joint motion of the knee joints of the lower limbs.^[7–10]^ However, it has been found that improved knee function in stroke patients is important in improving their walking function.^[11,12]^ Meanwhile, the Opti-Knee Motor Function Parameter is a test that does not restrict free movement of the trunk. It allows the test to be performed without any mechanical restrictions and provides a multi-degree-of-freedom, multi-axial view of the knee joint. Therefore, the aim of this study was to analyze the multi-axial joint angle changes of the healthy knee in stroke patients using the Opti-Knee knee function parameter instrument and to help clinicians and therapists to determine the basic characteristics of their gait and to provide a clinical basis for the next step of bilateral knee control training and improvement of walking ability in stroke patients.

## 2. Methods

### 2.1. Protocol and registration

This study was undertaken in strict accordance with the ethical principles of the Declaration of Helsinki for research in human medicine and approved by the Ethics Committee of Ningbo College of Health Sciences (Number: 2021-11-03). This study has been registered with the China Clinical Trials Registry (registration number: ChiCTR2100053702).

### 2.2. Design

The design was a cross-sectional study (pilot study).

### 2.3. Subjects

Sixty stroke patients who met the inclusion criteria in the rehabilitation ward of Ningbo Haishu Rehabilitation Hospital from December 2020 to December 2021 were selected as the observation group, and 60 normal people from the staff of Ningbo Haishu Rehabilitation Hospital and their family members who matched the age, height and weight of the patients in the control group were selected as the control group. All subjects participating in this study signed an informed consent form.

Inclusion criteria: Observation group: patients who met the 2018 Chinese guidelines for the diagnosis and management of acute ischemic stroke, confirmed by CT or MRI and diagnosed with cardiovascular disease^[13]^; aged 30 to 80 years; duration of disease ≥ 3 months, initial onset; all had a hemiplegic gait visible to the bare eyes, Holden walking function ≥ 2 levels; could walk continuously for more than 10m, and brunnstrom stage was 4 to 5; adapted to flat walking; voluntarily signed informed consent form.

Control group: healthy enough to walk naturally; no muscle, bone or joint disease of the lower limbs, no neurological disease or serious cardiopulmonary disease, normal movement of the joints of the lower limbs and feet; age 30 to 80 years.

Exclusion criteria: Observation group: patients with unstable vital signs, severe cardiopulmonary disease, severe cognitive impairment; sudden onset of a second stroke or other major illness at the time of testing; significant changes in blood pressure, heart rate or respiration at the time of testing (20% change from the baseline value or if the patient cannot tolerate it); patients who voluntarily give up in the middle of the test; patients with diseases of the healthy lower limb that cause walking impairment (osteoarthritis, etc).

Control group: patients with skeletal and muscular disorders of the lower limbs, neurological and cardiovascular diseases, psychological and psychiatric abnormalities, pregnant and lactating women, pathological gait, other knee pains and diseases that may contraindicate exercise, and those who have given up midway through the test.

**Figure 1.**
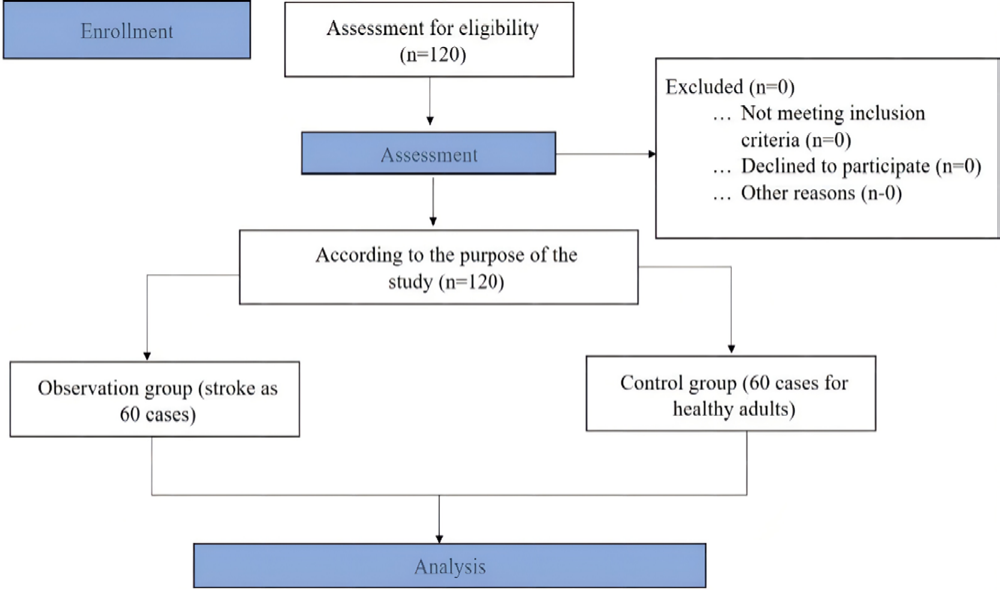
Study flow chart.

A total of 120 subjects meeting the above inclusion and exclusion criteria for the observation and control groups were enrolled in this study. According to the purpose of the study, they were divided into an observation group and a control group, with 60 stroke patients and 60 healthy adults each. At the same time, a test of the Opti-Knee tri-axial joint motion angle of the knee on the healthy side was completed for both groups of subjects. A final analysis was performed for the comparison. A flow chart of the study is shown in Figure 1.

### 2.4. Assessment

#### 2.4.1. Test method.

**Figure 2.**
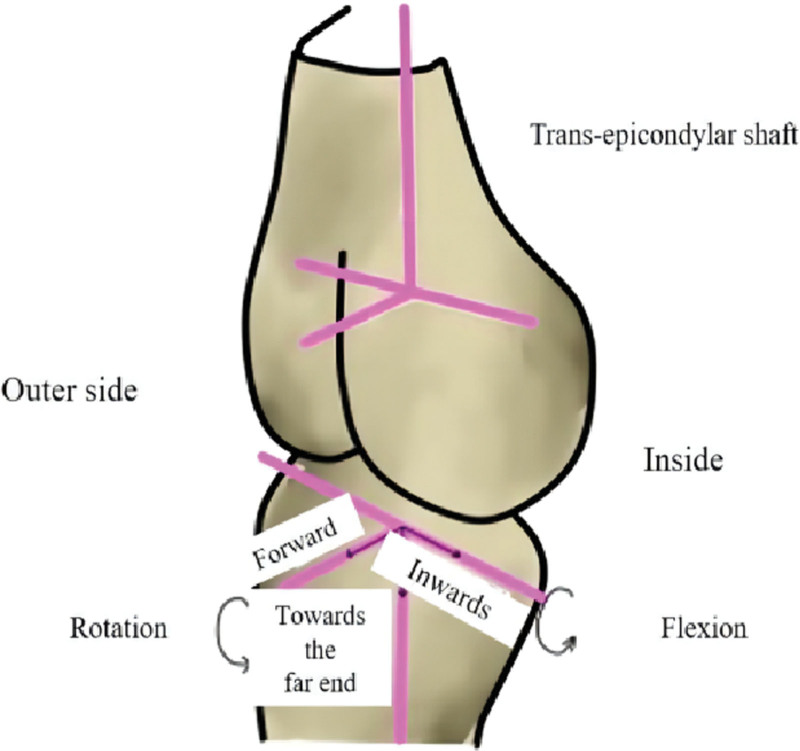
Degree of freedom of movement of the knee joint.

The Opti-Knee knee motion monitor (Model: YD-OK-694-DSP18) and its accompanying motion plate (Model: KF-205D) were used to mark the strapping components. All patients were subjected to the Opti-Knee Knee Kinetic Function Number Test to capture the motion of the knee joint during walking and to analyze the motion of the knee joint in 6 degrees of freedom during walking (See Fig. 2). The Opti-Knee Knee was used by 2 dedicated engineers for data acquisition and analysis.

**Figure 3.**
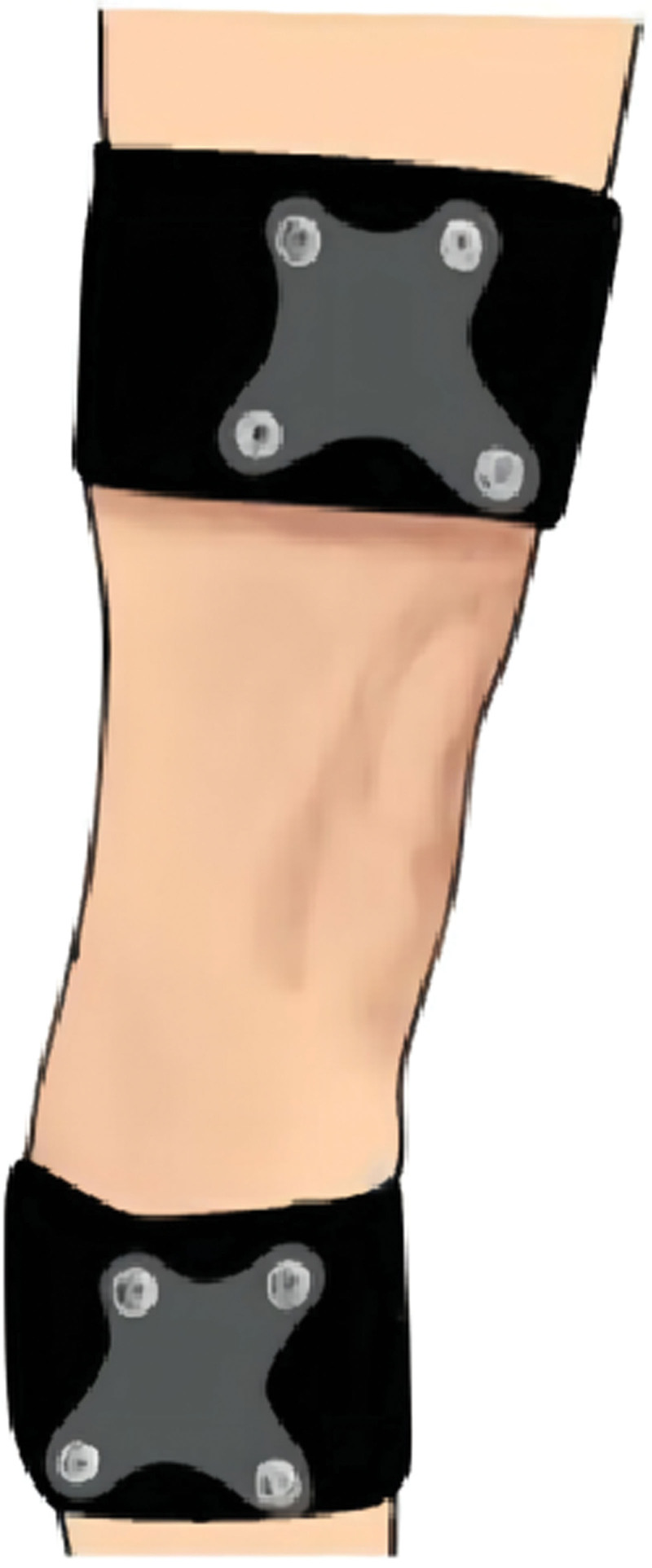
Illustration of restraints and stiffeners, tying.

**Figure 4.**
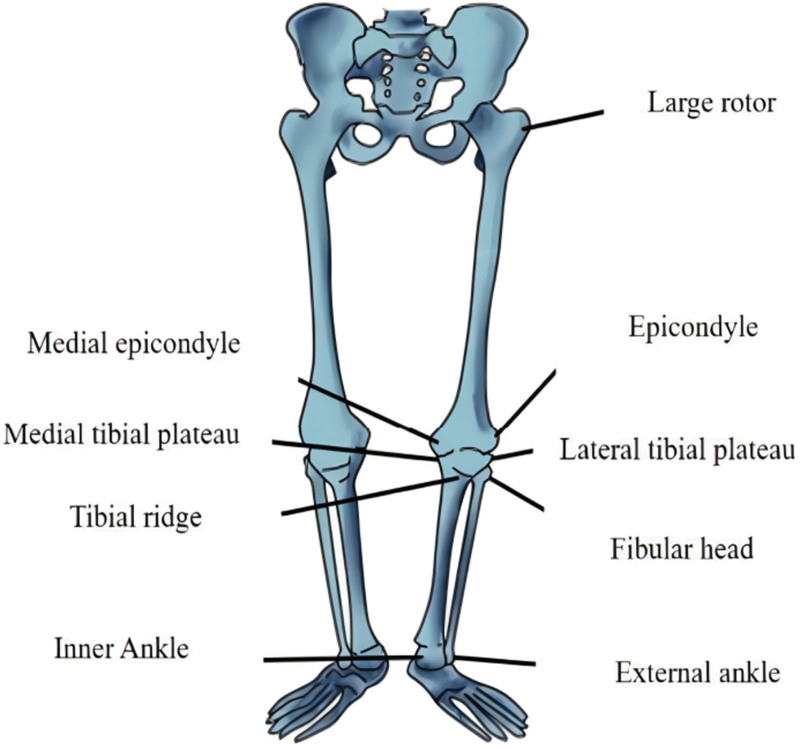
Schematic representation of the location of the osseous marker points.

**Figure 5.**
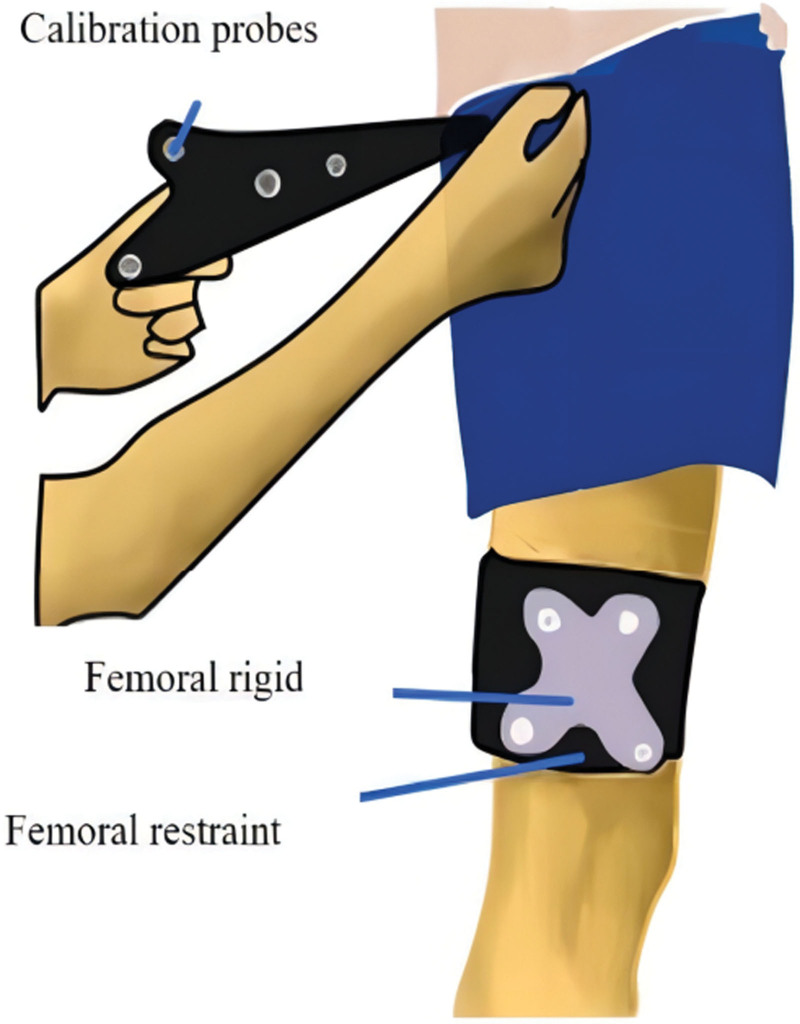
Schematic representation of the location of the osseous marker points.

Procedure^[14]^: Set up the Opti-Knee Knee Motion-Number Test and related equipment as required. The patient is placed on the exercise plate and the lower limb is fully exposed. Rigid body restraint: The tibial and femoral restraints are attached to the calf and thigh of the subject (the tibial and femoral restraints are generally positioned at the third of the limb near the knee joint), and the tibial and femoral restraints are used at the tibial and femoral restraints, respectively (See Fig. 3); Bone marker calibration: Touch the 9 individualized bone markers on the lower limb (see Fig. 4) and place the tracking probe at the bone markers (See Fig. 5), avoiding blocking any glow marks on the calibrator to prevent signal loss; Warm-up exercise: before the gait test the subject should choose a suitable pace (set at 0.8 km/h) on the treadmill for a few minutes to warm-up, so that the subject can walk in a normal position during the gait test and regain the subject’s true gait; Gait data extraction: the default acquisition time in the test software is 15 seconds, click the acquisition button, the software will instantly collect 15 seconds of the subject’s gait data. the tolerance of accuracy is ≤ +2, and the tolerance of displacement accuracy is ≤ ±2 mm.

#### 2.4..2. Assessment criteria.

The 6 degrees of freedom of the healthy knee and the control knee were measured in flexion (+), extension (−), maximum extension in the bracing phase and maximum flexion in the swing phase.^[14]^ The maximum extension angle in the bracing phase and the maximum flexion angle in the swing phase were assessed within 48 hours of entry. Definition of joint angles: in this study, a rigid body was tied around the knee joint and marked according to the characteristic points of the bony structures of the joint, then specific vector relationships were established. The movement of the knee joint was recorded in real-time by capturing the movement of the marker points on the surface of the body. The movement of the knee joint was calculated, and the movement of the joint was obtained in real-time with 6 degrees of freedom, that is, flexion/extension, external/internal rotation, internal/external rotation, anterior/posterior displacement, internal/external displacement and up/down displacement.

### 2.5. Statistical analysis

This study used Statistical Package for the Social Sciences vers.23.0 software for statistical analysis. The Kolmogorov-Smirnov normality test was used to compare all measures in the 2 groups. If the measures in the 2 groups conformed to a normal distribution, comparisons between groups were made using the independent samples *t* test and the data was expressed as mean ± standard deviation; if the measures in the 2 groups did not conform to a normal distribution, comparisons between groups were made using the *Wilcoxon* non-parametric rank-sum test and the data was expressed as median (quartile spacing). For comparisons of baseline data, for dichotomous data such as sex, stroke type and hemiplegic side, the *Chi-square* test was used and data was expressed as examples or percentages. The significant level was set at *P* < .05.

## 3. Results

### 3.1. General characteristics

A total of 120 eligible subjects were recruited for the study, comprising 43 males and 17 females in the observation group (n = 60) and the control group (n = 60). There were 39 males and 21 females in the control group, and the difference in gender between the 2 groups was demonstrated to be statistically insignificant (*P* > .05). No statistically significant differences were found in the comparison of subjects in both groups in terms of age, height, age, weight and body mass index (*P* > .05). No statistically significant differences were found in the comparison of subjects in the observation group in terms of duration of a stroke, type of stroke and side of hemiparesis (*P* > .05) (See Table 1).

**Table 1:** Baseline comparison of the general characteristics of the 2 groups.

	Observation group (n = 60)	Control group (n = 60)
Description of populations		
Gender		
M	43 (72%)	39 (65%)
F	17 (28%)	21 (35%)
Age (yrs)	56.14 ± 12.22	57.22 ± 13.62
Height (cm)	164.32 ± 7.84	166.35 ± 7.76
Weight (kg)	62.56 ± 5.62	63.43 ± 7.23
BMI	23.64 ± 2.57	27.61 ± 2.88
Time since stroke (mo)	5.61 ± 3.39	-
Type of stroke		
Ischemic	20 (33%）	-
Hemorrhagic	40 (67%）	-
Hemiplegic side (R/L)		
Right side	22 (37%)	-
Left side	38 (63%)	-

BMI = body mass index, F = female, L = left, R = right, M = male.

### 3.2. Comparison of knee joint movement angles, maximum extension and flexion angles

Compared to the control group, the changes in the knee joint movement angles of flexion and extension, internal and external rotation and internal and external turning were statistically significant [95%(37.22, 45.13), *P* = .01], [95%(9.51,13.67), *P* = .018], [95%(4.82,7.57), *P* = .049]. At the same time, the changes in maximum extension and flexion angles were statistically significant in the observation group [95%(4.12, 8.63), *P* = .019], [95%(51.68, 57.28), *P* = .0001] (See Table 2).

**Table 2:** Comparison of knee motion angles, maximum extension and flexion angles between the 2 groups.

Index	Observation group (n = 60)	Control group (n = 60)	(95% CI)	*P* value
Flexion and extension	41.17 ± 11.51	58.85 ± 3.71	(37.22, 45.13)	.01
Internal and external rotation	11.59 ± 6.05	13.57 ± 3.81	(9.51,13.67)	.018
Internal and external turning	6.19 ± 3.99	8.29 ± 3.10	(4.82,7.57)	.049
Maximum angle of extension	6.77(3.00–14.12)	3.37(−0.59 to 5.97)	(4.12, 8.63)	.019
Maximum angle of flexion	47.86(42.71–55.50)	63.03(60.62–65.31)	(51.68, 57.28)	.0001

**Figure 6.**
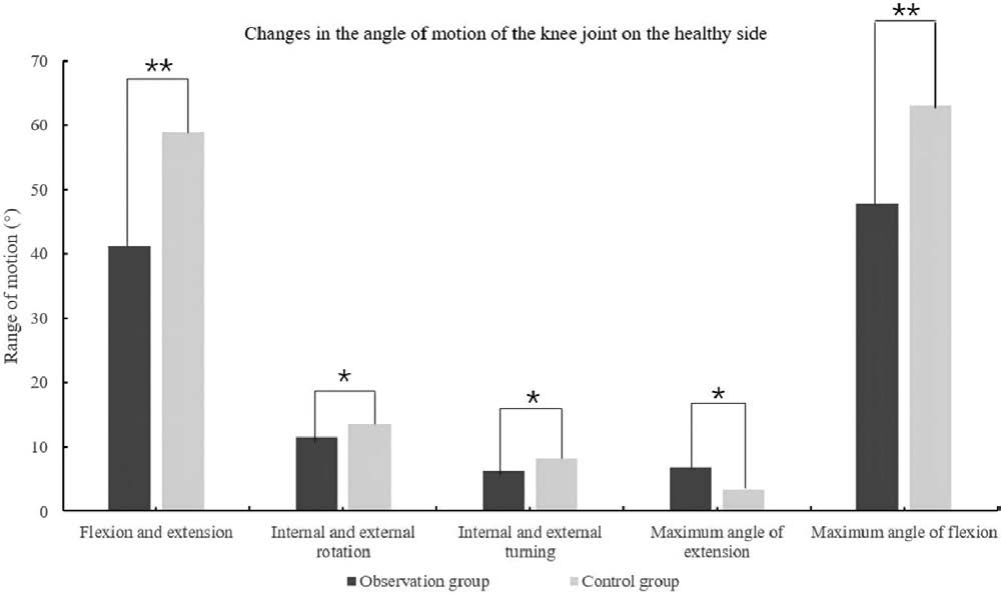
Changes in the angel of motion of the knee on the health side. Note: **The difference is highly significant, *Significantly different.

As seen in Figure 6, the observation group revealed the most significant trend in knee flexion and extension angle and maximum flexion angle. However, although the difference between the 2 groups in the comparison of internal and external rotation, internal and external turning and maximum extension angle of the knee joint was statistically significant, its trend of change was not significant.

### 3.3. Comparison of knee joint displacement angles

**Figure 7.**
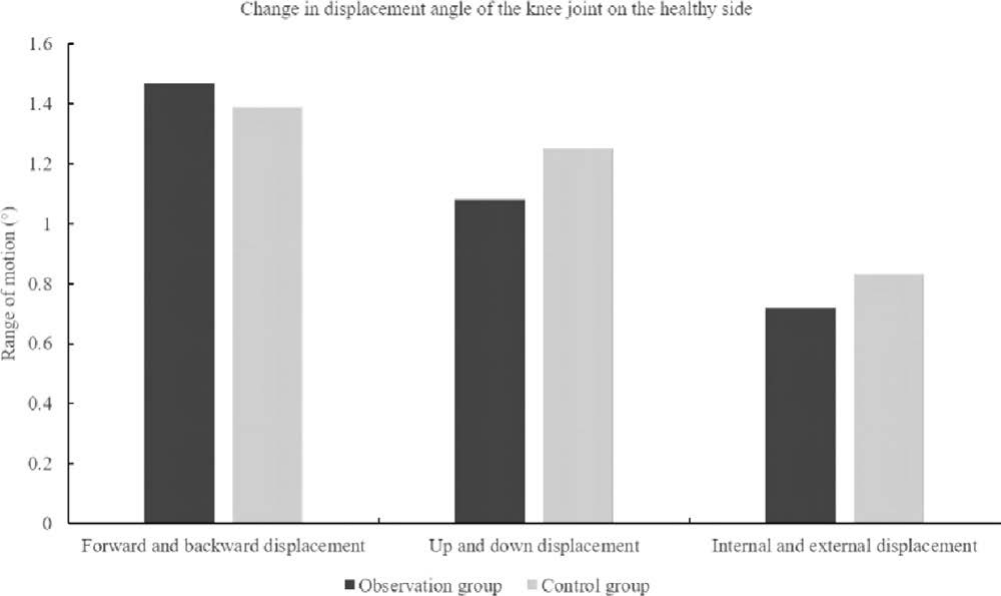
Changes in displacement angle of the knee joint on the health side.

Compared to the control group, there was no significant change in the forward-backward, up-down and internal-external displacement angles of the knee in the observation group, and the difference was not statistically significant [95%(1.16, 1.78), *P* = .72], [95%(0.85,1.32), *P* = .32], [95%(0.57, 0.88), *P* = .36] (See Table 3). As seen in Figure 7, there was no significant change in forward-backward, up-down and internal-external displacement of the knee joint in both groups.

**Table 3:** Comparison of knee joint displacement angles between the 2 groups.

Index	Observation group (n = 60)	Control group (n = 60)	(95% CI)	*P* value
Forward and backward displacement	1.47 ± 0.89	1.39 ± 0.46	(1.16, 1.78)	.72
Up and down displacement	1.08 ± 0.68	1.25 ± 0.39	(0.85,1.32)	.32
Internal and external displacement	0.72 ± 0.44	0.83 ± 0.31	(0.57, 0.88)	.36

## 4. Discussion

This study was conducted to observe the joint mobility in multiple axes of the knee joint in stroke patients bilaterally to better understand the factors affecting knee stability so as to provide a practical clinical basis for the next step of rehabilitation training. The study confirmed that the range of motion of flexion and extension, internal and external rotation, internal and external turning, maximum extension and maximum flexion angles of the knee on the healthy side were significantly reduced in the observation group. This means that the angle of flexion and extension, internal and external rotation, internal and external turning, maximum flexion and extension of the knee on the healthy side may affect the stability of their knee and their improved walking ability.

Previous assessments of walking function in stroke patients have focused on the function of the lower limb on the affected side, particularly the hip and ankle. Less attention has been paid to the movement of the “healthy knee.” The knee is a flexor joint and can also perform mild gliding and rotation in flexion.^[15,16]^ Its function is to bear weight, transmit loads and provide function for the movement of the lower leg.^[17]^ The aim of this study was thus to capture the movement of the “healthy knee” in the abnormal stroke gait and to compare it with the movement of the true healthy knee in normal gait to understand what abnormal movements are present in the “healthy knee” of patients with stroke walking dysfunction.

Wang et al conducted a kinematic comparison between the healthy and normal bilateral lower limbs of stroke patients and found significant differences in tempo/ral and spatial parameters between the healthy and normal bilateral lower limbs, and analyzed that the reason for this difference was the presence of a functional compensatory effect of the healthy side on the affected limb.^[18]^ The results of this study found that the range of motion of the flexion and extension angle, internal and external rotation and internal and external turning of the knee on the healthy side of the observation group was significantly smaller than that of the control group. This may be since during the swing phase of the lower limb on the affected side during the support phase of hemiplegic gait in stroke, the knee joint cannot be flexed in a coordinated manner due to the increased muscle tone of the extensor and abductor muscles of the affected side of the lower limb, while the dorsiflexion of the ankle joint is restricted and the foot drops, preventing the foot from leaving the ground adequately. The body’s center of gravity is severely skewed, and the body is unstable; the knee on the healthy side is then moved passively to provide stable support for the body.^[19–23]^

The results of this study confirmed that the maximum extension and maximum flexion angles of the healthy side of the knee were less than normal in the observation group of stroke patients, with the change in maximum flexion angle being more prominent than normal. This may be because the lower limb on the affected side is weakly supported by weight-bearing, resulting in a shorter single-support period, and the swing speed of the healthy lower limb is accelerated, so that to shorten the swing time of the healthy lower limb, the knee on the healthy side may reduce its activity in order to enter the support period quickly.^[24,25]^ to compensate for the lack of function of the affected lower limb when walking, the stroke patient relies more on the healthy lower limb to maintain balance and walk, causing the healthy lower limb to carry more of the body load.^[25,26]^

In summary, if a stroke patient is severely weight-bearing on the right side, this may lead to a reduction in joint space, causing joint wear and tears and pain. As the structure of the knee joint changes in the long run it can influence the normal position of the pelvis, hip and ankle, and the body’s center of gravity changes, affecting balance and increasing the risk of falls. This effect may be more apparent if the lower limb on the affected side is not able to flex the knee properly during the swing phase, leading to pain in the healthy knee in many patients, which in turn affects walking ability and walking stability.^[27,28]^ Jiang etc showed that the initial moment of landing, maximum extension angle in the standing phase, maximum flexion angle in the swing phase, and sagittal knee angle range of the affected and healthy knee joints in stroke patients were significantly different from those of normal subjects.^[19]^ This is consistent with the results of this study and again indicates that the Opti-knee Knee Motion Detection System is effective in observing changes in knee motion angles in all axes.^[14]^

There are also certain limitations to this study. Firstly, only the change in joint angle in the 3 axes of the knee joint on the healthy side was observed, and the change in motion of the trunk, hip and knee joints was not compared to the normal population. Further consideration of the effect of overall postural changes on walking ability is needed in subsequent studies. Secondly, this study did not consider the effect of changes in plantar pressure on the stability of the knee joint on the healthy side, as changes in plantar pressure can represent the stresses experienced by stroke patients during standing and walking and are important for further analysis of gait. Thirdly, in the initial design, it was envisioned that surface electromyography changes in the quadriceps, shrimps and internal thigh muscle groups would be included in the test. Due to equipment failure, this resulted in their muscle changes during walking not being effectively reacted to. However, this study also innovatively made a multidimensional and comprehensive observation of the joint angle changes in the knee joint on the unaffected side of stroke patients, which laid the foundation for subsequent correction of abnormal posture and gait.

## 5. Conclusion

The results of this study confirm that changes in the angle of motion of the knee on the side of the stroke are important in preserving knee stability. As further research is conducted, we should not overlook the importance of the stroke patient’s healthy knee in overall postural control and walking. Only by properly addressing the importance of the stroke patient’s healthy knee can we reduce the abnormal compensatory patterns of stroke patients and train them the correct walking patterns. This will lay the foundation for enhanced overall postural control and walking ability.

## Author contributions

**Conceptualization:** JunWu Yu, FaMing Yang.

**Data curation:** Chen Wang, JunWu Yu.

**Formal analysis:** Chen Wang, JunWu Yu, FaMing Yang.

**Funding acquisition:** FaMing Yang.

**Investigation:** Chen Wang, JunWu Yu, FaMing Yang.

**Methodology:** FaMing Yang.

**Project management:** FaMing Yang.

**Resources:** FaMing Yang.

**Software:** JunWu Yu.

**Supervision:** FaMing Yang.

**Validation:** FaMing Yang.

**Visualization:** Chen Wang, JunWu Yu.

**Writing – original draft:** JunWu Yu.

**Writing – review & editing:** Chen Wang, JunWu Yu, FaMing Yang.
